# Molecular evidence of potential novel spotted fever group rickettsiae, *Anaplasma* and *Ehrlichia* species in *Amblyomma* ticks parasitizing wild snakes

**DOI:** 10.1186/s13071-015-0719-3

**Published:** 2015-02-19

**Authors:** Kai Ling Kho, Fui Xian Koh, Sun Tee Tay

**Affiliations:** Department of Medical Microbiology, Faculty of Medicine, University of Malaya, Lembah Pantai, 50603 Kuala Lumpur, Malaysia

**Keywords:** *Amblyomma* ticks, *Rickettsia raoultii*, *Rickettsia tamurae*, Malaysia

## Abstract

**Background:**

*Amblyomma* ticks parasitize a wide range of animals in tropical regions. This study describes the identification of *Amblyomma* ticks from wild snakes in Malaysia and the detection of potential human pathogens such as *Rickettsia*, *Anaplasma, Ehrlichia* and bartonellae in the ticks.

**Findings:**

Twenty one adult ticks (twelve *A. varanense* and nine *Amblyomma helvolum* ticks) identified from seven *Python molurus* snakes in Sepang and a pool of six *A. helvolum* ticks from a *Naja sumatrana* snake in Johore, Malaysia were investigated in this study. Amplification of the citrate synthase (*gltA*), 190-kDa surface antigen gene (*ompA*), 135-kDa surface antigen (*ompB*) and surface cell antigen (*sca4*) genes followed by sequence analysis confirmed the presence of two potential novel spotted fever group rickettsiae in the ticks. *Candidatus* Rickettsia sepangensis from an engorged *A. varanense* tick demonstrated high sequence similarity to *Rickettsia tamurae*; while *Candidatus* Rickettsia johorensis from two samples (individual and pooled) of *A. helvolum* and two *A. varanense* ticks were closely related to *Rickettsia raoultii. Anaplasma* and *Ehrlichia* DNA were detected from seven and two ticks, respectively. No bartonellae was detected from any of the ticks.

**Conclusion:**

The finding in this study suggests that *Amblyomma* ticks parasitizing wild snakes may serve as reservoir hosts and carriers for rickettsioses, anaplasmosis and ehrlichiosis in this region.

## Background

Ticks are the vector for numerous emerging zoonotic diseases which can be severe and life-threatening to humans. In nature, ticks and a wide range of animals may act as reservoirs or amplifiers for human pathogens such as spotted fever group rickettsiae, anaplasma, ehrlichiae and bartonellae. Humans can be accidentally infected with these organisms through tick bites. The ticks belonging to the genus *Amblyomma* have been implicated as a carrier for several pathogenic rickettsiae including *Rickettsia rickettsii*, *R. aeschlimannii, R. raoultii*, and *R. tamurae* [1], *Anaplasma phagocytophilum*, *Ehrlichia chaffeensis* and *E. ewingii* [2,3]. Additionally, *Bartonella* DNA has also been detected in *A. americanum* ticks [4].

*Amblyomma* ticks parasitize a wide range of animals and are often seen on mammalian hosts, reptiles and amphibians [5,6]. However, information is lacking on tick carriage of emerging human pathogens in the tropical region. In this study, we assessed the occurrence of these microorganisms in *Amblyomma* ticks parasitizing wild snakes in Malaysia by using molecular approach.

## Methods

Twenty-one adult ticks (12 *A. varanense* and nine *A. helvolum*) from seven *Python molurus* snakes from Sepang (2°49′10.862″N, 101°44′1.262″E) and a pool of six *A. helvolum* ticks from a Spitting cobra (*Naja sumatrana*) in Johore, Malaysia (1°43′58.321″N, 103°54′5.082″E) collected from August-October 2012 were investigated in this study. The ticks were identified based on the taxonomic keys of Burridge [5] and Kohls [7].

Tick DNA was extracted using QIAamp DNA mini kit (Qiagen, Hilden, Germany) in accordance to the manufacturer’s instruction. Four rickettsial-specific genes were targeted for amplification from the tick samples, i.e., citrate synthase gene (*gltA*), 190-kDa outer membrane protein gene (*ompA*), 135-kDa outer membrane protein gene (*ompB*) and surface cell antigen (*sca4*) [8-11]. Identification of *Anaplasma* and *Ehrlichia* DNA in the samples was performed using a PCR assay targeting 16S rRNA gene of the organisms [12] followed by sequence analysis. For further differentiation of *Anaplasma* spp., amplification of the full length sequences of 16S rDNA and *msp4* genes were performed [13]. A PCR assay targeting citrate synthase (*gltA*) gene was performed for detection of bartonellae DNA [14]. Cloned PCR2.1-TOPO T/A plasmids (Invitrogen, USA) with amplified *gltA* fragment from *R. honei* (strain TT118), *ompA* and *ompB* fragments from rickettsial endosymbionts (98% similarity to *R. heilongjiangensis* and *R. raoultii,* respectively) of tick samples were used as positive controls. BLAST analysis was performed to search for homologous sequences in the GenBank database. To determine the phylogenetic position of the rickettsiae identified in this study, dendrogram was constructed based on concatenated sequences of *gltA* (1040–1046 nucleotides) and *ompA* (407–431 nucleotides) genes using neighbour-joining method of MEGA software [15].

## Findings

Table 1 shows the amplification of rickettsial *gltA* gene from three *A. varanense* (S5, S4-2 and S7-2) and two *A. helvolum* tick samples (S6-1, P1). The *gltA* and *ompA* sequences from the S5 tick was almost similar (99.0% and 97.7%, respectively) with *R. tamurae* strain AT-1 from *A. testudinarium* tick in Japan [16]. However, the *ompB* gene of the rickettsia was unable to be amplified and no significant similarity was obtained for the amplified *sca4* fragment.

**Table 1:** **Molecular detection of rickettsiae, anaplasma and ehrlichia and blast analysis of the sequences derived from tick samples in this study**

**Tick sample (Species, location)**	***Rickettsia***				***Anaplasma 16S rDNA***
	***gltA***	***ompA***	***ompB***	***sca4***	
***Candidatus*** **Rickettsia sepangensis**					
S5(*A. varanense*, Sepang)	*R. tamurae* strain AT-1 (AF394896) (1033/1043, 99.0%)	*R. tamurae* strain AT-1 (DQ103259) (417/427, 97.7%)	Unable to be amplified	No significant similarity	*A. phagocytophilum* (AY551442, 99%, 253/256), *A. platys* (JX261979, 99% 253/256)
***Candidatus*** **Rickettsia johorensis**					
P1 (pooled *A. helvolum*, Johore), S4-2 (*A. varanense*, Sepang), S6-1 (*A. helvolum,* Sepang)	*R. raoultii* strain Khabarovsk (DQ365804) (1057/1060, 99.7%)	*R. raoultii* strain Khabarovsk (DQ365801) (418/429, 97.4%)	*R. raoultii* strain Khabarovsk (DQ365798) (762/775, 98.3%)	*R. raoultii* strain Khabarovsk (DQ365808) (795/816, 97.4%)	Not amplified
S7-2 (*A. varanense*, Sepang)	*R. raoultii* strain Khabarovsk (DQ365804) (1057/1060, 99.7%)	*R. raoultii* strain Khabarovsk (DQ365801) (418/429, 97.4%)	*R. raoultii* strain Khabarovsk (DQ365798) (762/775, 98.3%)	*R. raoultii* strain Khabarovsk (DQ365808) (795/816, 97.4%)	*A. bovis* (AB983438, 99%, 253/256)
S2, S4 (*A. helvolum,* Sepang), S6, S7 (*A. varanense*, Sepang)	Not amplified				*A. phagocytophilum* (AY551442, 99%, 253/256), *A. platys* (JX261979, 99%, 253/256)
S6-2 (*A. varanense*, Sepang)	Not amplified				*A. bovis* (AB983438, 99%, 253/256)
S3, S7-3 (*A. varanense*, Sepang)	Not amplified				*Ehrlichia* spp. (J410257, 99%, 249/256)

The sequences obtained for rickettsiae from S5 and P1 ticks have been deposited in the GenBank database under the accession numbers: [*gltA* (GenBank: KJ769648, KJ769650), *ompA* (GenBank: KJ769649, KJ769651), *ompB* (GenBank: KJ769652), *sca4* (GenBank: KM977711)].

BLAST analysis of the rickettsial *gltA* sequence from two samples (individual and pooled) of *A. helvolum* (S6-1, P1) and two *A. varanense* (S4-2 and S7-2) ticks demonstrated the closest match (99.7%) to *R. raoultii* strain Khabarovsk (Table 1), which was cultivated from *Dermacentor* ticks in Russia and France [17]. The sequence similarity of the *ompA, ompB* and *sca4* sequences of these ticks with those of *R. raoultii* strain Khabarovsk was 97.4%, 98.3% and 97.4%, respectively.

**Figure 1:**
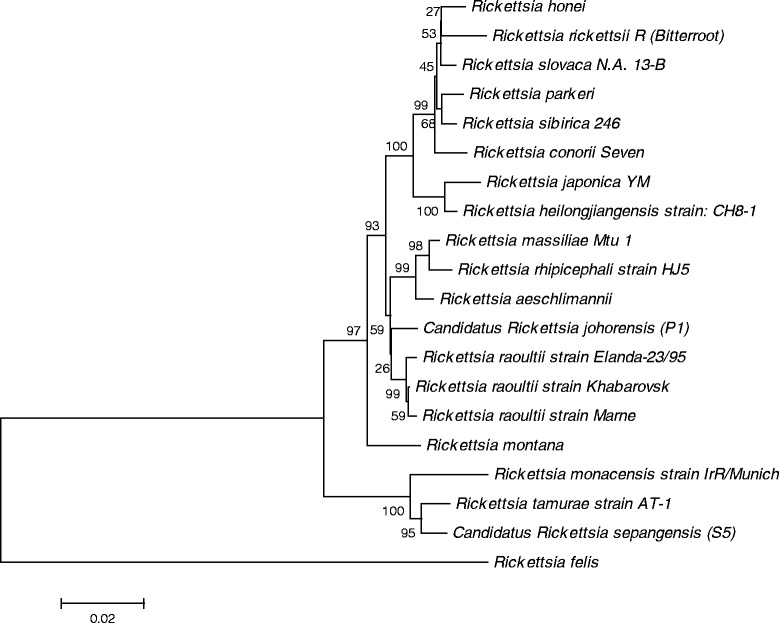
**Phylogenetic placement of concatenated sequences (** ***gltA*** **and** ***ompA*** **) of known rickettsial species in Table** **2** **.** Bootstraps analysis was performed with 1000 replications. Numbers in brackets are GenBank accession numbers. Scale bar indicates the nucleotide substitutions per sites.

According to the current criteria for speciation of rickettsial species, uncultured rickettsia exhibiting sequence similarity of ≤99.9% for *gltA*, ≤ 98.8% for *ompA*, ≤99.2% *ompB* and ≤99.3% for *sca4* genes with a validated *Rickettsia* species may be given *Candidatus* status [18]. Hence, the rickettsiae are thus named as *Candidatus* Rickettsia sepangensis and *Candidatus* Rickettsia johorensis, respectively, in accordance to the location of their first sample collection. The dendrogram constructed using concatenated sequence of *gltA* and *ompA* gene fragments (Table 2 and Figure 1) confirmed the clustering of *Candidatus* Rickettsia sepangensis with the type strain of *R. tamurae*, and *Candidatus* Rickettsia johorensis with *R. raoultii* type strains.

**Table 2:** **GenBank accession numbers of the rickettsial gene sequences used for the construction of a concatenated NJ tree**

***Rickettsia*** **sp.**	**GenBank accession no. for targeted genes**	
	***gltA***	***ompA***
*Rickettsia raoultii* strain Elanda-23/95	EU036985	EU036986
*Rickettsia raoultii* strain Khabarovsk	DQ365804	DQ365801
*Rickettsia raoultii* strain Marne	DQ365803	DQ365799
*Rickettsia aeschlimannii*	AY259084	AY259083
*Rickettsia massiliae* Mtu 1	U59719	U43799
*Rickettsia rhipicephali* strain HJ5	DQ865206	DQ865208
*Rickettsia parkeri*	KF782319	KF782320
*Rickettsia sibirica* 246	U59734	U43807
*Rickettsia conorii* Seven	U59730	U43806
*Rickettsia honei*	AF018074	AF018075
*Rickettsia rickettsii* R (Bitterroot)	U59729	U43804
*Rickettsia montana*	U74756	U43801
*Rickettsia tamurae* strain AT-1	AF394896	DQ103259
*Rickettsia japonica* YM	U59724	U43795
*Rickettsia heilongjiangensis* strain CH8-1	AB473812	AB473813
*Rickettsia felis* strain URRWXCal2	AF210692	AF210694
*Rickettsia slovaca* N.A. 13-B	U59725	U43808
*Rickettsia monacensis* strain IrR/Munich	DQ100163	DQ100169
*Candidatus* Rickettsia sepangensis (S5)	KJ769648	KJ769649
*Candidatus* Rickettsia johorensis (P1)	KJ769650	KJ769651

Several spotted fever group rickettsiae with unknown or potentially pathogenicity for humans have been reported in the Southeast Asia region, mainly in Thailand. *R. honei* (strain TT-118) and *R. thailandii* sp. nov. have been identified from *Ixodes* and *Rhipicephalus* ticks [19,20]. Closely related species of *R. raoultii* have also been detected from *A. helvolum* from a lizard (*Varanus salvator*) in Thailand [21]. Exposure to infected snake ticks may pose risks to human health as *R. tamurae* and *R. raoultii* have been implicated in human infections [22,23]. High antibody prevalence to *R. honei* (TT118 strain) has been reported in febrile patients in rural areas in Malaysia [24]. However, information on the type of spotted fever group rickettsiae is still lacking.

*Anaplasma* DNA was amplified from seven ticks (Table 1). Based on the 256 nucleotides of the amplified 16S rDNA partial gene fragments, sequences from three *A. varanense* and two *A. helvolum* ticks showed the closest similarity to those of *A. phagocytophilum* [Genbank accession no.: AY551442, 99%, 253/256] or *A. platys* [Genbank accession no.: JX261979, 99%, 253/256]. *A. bovis* DNA [Genbank accession no.:AB983438, 99%, 253/256] was amplified from two *A. varanense* ticks, whereas DNA of *Ehrlichia* spp. [Genbank accession no.: KJ410257, 99%, 249/256] was amplified from two *A. varanense* ticks. Attempts to determine the full length sequence of 16S rRNA and *msp4* genes were not successful as the sequences obtained were not satisfactory for analysis. No bartonellae was detected from any of the ticks understudied.

There is no report on the human infections caused by tickborne pathogens with reptile as a host in Southeast Asia. The presence of SFG rickettsiae (*Rickettsia* species closely related to *R. raoultii*, *R. tamurae* and *R. bellii*) has been recently shown in *A. varanense* and *A. helvolum* in Thailand [25]. Detection of *R. honei* in a reptilian tick, *Bothriocroton hydrosauri* (formerly *Aponomma hydrosauri*) has been reported in Australia [26]. *Rickettsia* spp. closely related to *R. tamurae* has also been detected in *A. fimbriatum* ticks collected from reptiles (yellow-spotted monitor, water python and green-tree snake) in the Northern Territory of Australia [27], and *A. exornatum* tick from a lizard (*Varanus olivaceus)* in United States of America [28]. In the South America, *Rickettsia* sp. strain Colombianensi has been identified from *A. dissimile* ticks parasitizing iguanas in Colombia [29]. All these findings suggest the existing of a natural cycle of spotted fever group rickettsial infection in ticks and snakes in different geographical regions. *A. phagocytophilum* has been detected in *A. flavomaculatum* tick collected from a *Varanus exanthematicus* lizard imported into Poland [30]. Meanwhile, the detection of *Ehrlichia* spp. from ticks collected from snakes has not been reported previously and thus, merits further investigation.

*A. helvolum* ticks have been identified from different snakes including *Python* sp., *Ptyas* (Zamensis) *korros* and *Naja naja* (Kohls) [7] in Malaysia. *A. varanense* is also one of the most widespread *Amblyomma* ticks in large snakes in Southeast Asia [5]. As *P. molurus* and *N. sumatrana* snakes are native to Southeast Asia [31,32], ticks parasitizing the snakes could be endemic where the animal hosts are available. Although there is no data about the affinity of the ticks to bite humans yet, the detection of rickettsial agents in the snake ticks poses a risk to both wildlife and human. Further work is required to assess the prevalence of these potential tick-borne pathogens on a larger scale.

## Conclusions

This study presented the molecular evidence of the presence of potential novel spotted fever group rickettsiae closely related to *R. tamurae* and *R. raoultii*, *Anaplasma* and *Ehrlichia* spp. in two species of *Amblyomma* ticks parasitizing *P. molurus* and *N. sumatrana* snakes. The finding in this study suggests the potential role of *Amblyomma* ticks as a reservoir host and carrier for rickettsioses, anaplasmosis and ehrlichiosis in this region.
